# Mechanochemistry and oleochemistry: a green combination for the production of high-value small chemicals

**DOI:** 10.3389/fchem.2023.1306182

**Published:** 2023-11-27

**Authors:** Christophe Len, Vaishaly Duhan, Weiyi Ouyang, Remi Nguyen, Bimlesh Lochab

**Affiliations:** ^1^ School of Chemistry, Xi’an Jiaotong University, Xi’an, China; ^2^ Institute of Chemistry for Life and Health Sciences, Chimie ParisTech, PSL Research University, Paris, France; ^3^ Materials Chemistry Laboratory, Department of Chemistry, School of Natural Sciences, Shiv Nadar Institution of Eminence, Gautam Buddha Nagar, Greater Noida, India

**Keywords:** mechanochemistry, vegetable oil, glycerol, batch reactor, continuous flow reactor

## Abstract

Mechanochemistry and oleochemistry and their combination have been known for centuries. Nevertheless, bioeconomy and circular economy concepts is much more recent and has motivated a regain of interest of dedicated research to improve alternative technologies for the valorization of biomass feedstocks. Accordingly, this review paper aims essentially at outlining recent breakthroughs obtained in the field of mechanochemistry and oleochemicals such as triglycerides, fatty acids, and glycerol derivatives. The review discusses advances obtained in the production of small chemicals derived from oils with a brief overview of vegetable oils, mechanochemistry and the use of mechanochemistry for the synthesis of biodiesel, lipidyl-cyclodextrine, dimeric and labelled fatty acids, calcium diglyceroxide, acylglycerols, benzoxazine and solketal. The paper also briefly overviews advances and limits for an industrial application.

## 1 Introduction

For several decades now, society has been confronting significant challenges directly linked to our way of life. The world’s population continues to grow, leading to an ever-increasing demand for energy. At the same time, concerns arise due to the scarcity of resources, including food and water, and the depletion of fossil fuel reserves. These issues are of utmost importance for the wellbeing of future generations. To address these challenges, scientists, governments, politicians, and citizens are actively engaged in creating and promoting new practices to establish a more sustainable society for the future. However, achieving this societal transformation is no simple task and demands substantial, multidisciplinary collaborative efforts to develop greener technologies and environmentally friendly methodologies such as mechanochemistry (Mateti et al., 2021; Michalchuk et al., 2021; Bolt et al., 2022; Cuccu et al., 2022), microwave chemistry (da Costa et al., 2023; Kumar et al., 2023; Nguyen et al., 2021; Zhao et al., 2020; Rodriguez-Padron et al., 2020), sonochemistry (Maddikeri et al., 2012; Martinez et al., 2021; Kargar et al., 2022) and others. Mechanochemistry, a cutting-edge branch of chemistry, aligns seamlessly with the principles of “green chemistry.” Green chemistry aims to design and develop chemical processes that are more environmentally friendly, sustainable, and less hazardous. Mechanochemistry contributes to these goals by promoting efficient, solvent-free, and energy-saving reactions. It eliminates or minimizes the use of toxic or harmful reagents and reduces waste generation. Additionally, mechanochemical processes often require lower temperatures, lowering energy consumption and greenhouse gas emissions. The technique also enhances reaction selectivity, leading to fewer byproducts and less need for purification steps. This synergy between mechanochemistry and green chemistry fosters innovation in the development of cleaner, more sustainable chemical processes. It opens the door to more eco-friendly manufacturing, benefiting both the environment and the chemical industry, making it a crucial component of sustainable chemistry practices. Among the promising alternatives to tackle future challenges are renewable resources such as vegetable oil (Xia and Larock, 2010; Yara-Varon et al., 2017), lignocellulose (Vu et al., 2020; Lobato-Peralta et al., 2021; Ning et al., 2021), and others and the concept of biorefinery (Botero Gutierrez et al., 2017; Calvo-Flores and Martin-Martinez, 2022). Oleochemistry, the chemistry of oil plays a pivotal role in harnessing renewable resources for various applications. Derived from natural fats and oils, oleochemicals serve as a sustainable alternative to traditional petrochemicals. This branch of chemistry is centered around utilizing triglycerides and fatty acids from plant and animal sources, making it inherently renewable. One of the key advantages of oleochemistry is its reliance on bio-based feedstocks, which are replenishable through agriculture and farming practices. This reduces our dependence on finite fossil resources, contributing to a more sustainable and eco-friendly chemical industry. Oleochemicals can be transformed into a wide range of valuable products, including biodiesel, soaps, surfactants, lubricants, and more. Furthermore, oleochemistry aligns with the principles of green chemistry, emphasizing the use of environmentally benign processes and reduced waste generation. It offers a viable pathway to reduce greenhouse gas emissions and mitigate environmental impacts associated with traditional petrochemical production. As the world seeks more sustainable alternatives, oleochemistry stands as a promising and renewable resource with a bright future in various industries. By adopting processes similar to or even more sustainable than those used in petrochemicals, we can effectively recover biomass and biomass waste (da Costa et al., 2022; Khodadadi et al., 2020), enabling the production of valuable chemicals, materials, fuels, and energy (Nayak and Bhushan, 2019; Chen et al., 2020; Nasrollahzadeh et al., 2020). Despite the advantages, the use of biomass also presents certain challenges. One such issue is the competition between food/feed production and non-food chemistry, as the demand for biomass increases. Additionally, the expanded use of biomass has implications for land use and water availability, necessitating careful consideration and sustainable management practices.

Nevertheless, the chemical industry has a growing need to replace petro-sourced small molecules with bio-sourced molecules and, also to create new biobased molecules with identical or different properties using green innovative technology. This review aims to present and comment on the latest advancements in the valorization of oils such as triglycerides, fatty acids, fatty esters, and glycerol (oleochemistry) through mechanochemistry as an innovative technique for the production of high-value small molecules.

## 2 Vegetable oils: production, chemical modification to small chemicals

According to the US Department of Agriculture (USDA), the world’s vegetable oil production for the year 2023/24 is projected to reach 222.8 million tonnes, showing a 2.7% increase compared to the previous year (2022/2023) (Vegetable oil production in 2023/24 expected to be up on previous year, 2023). In 2022, the Global Vegetable Oil Market was valued at USD 2.192 Billion, and it is expected to grow significantly with a projected value of USD 4.46 Billion by 2030. The industry is expected to register a Compound Annual Growth Rate (CAGR) of 9.3% during the forecast period of 2022–2028 (Vegetable oil market–Industry analysis, trends and forecast (2023-2029). The global vegetable oil market is segmented based on type, including soybean oil, rapeseed oil, sunflower oil, palm oil, olive oil, corn oil, peanut oil, coconut oil, and others. Furthermore, it is categorized by packaging applications, with food products accounting for 27%, followed by animal feed at 21%, cosmetics at 19%, pharmaceuticals at 13%, biodiesel at 10%, and others at 10%. The distribution channels include direct sales, supermarkets, convenience stores, specialty stores, e-commerce, and others. Key players in this market include Cargill, Wilmar International, Archer Daniels Midland Company, Avril group, and others (Vegetable oil market–Industry analysis, trends and forecast (2023-2029).

**FIGURE 1:**
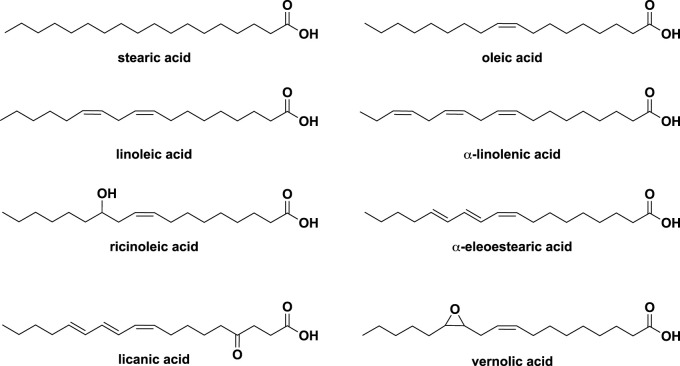
Main fatty acids present in the composition of vegetable oils.

The main components of vegetable oils are triglycerides, which consist of a glycerol moiety and three fatty acid chains, either identical or different. Figure 1 illustrates the most common natural fatty acids that have been identified for the vegetable oils.

**FIGURE 2:**
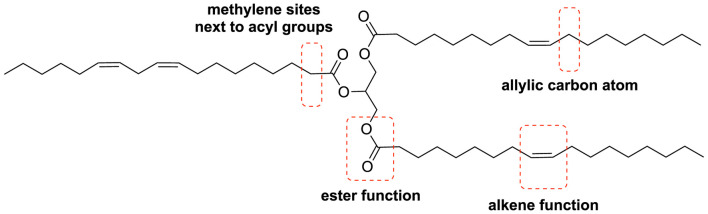
Main chemical reactive sites of unsaturated triglycerides.

Most vegetable oils are utilized either directly or shortly after refinement, but some require chemical modification before they can be used. This modification process involves various chemical reactions targeting the carboxy groups, the methylene sites next to ester, the unsaturated bonds or the allylic carbon atom, tailored to meet specific industrial requirements (Figure 2). In the case of ricinolein, another possibility is to modify the hydroxyl group in position 12.

In industrial processing, vegetable oils are transformed into glycerol, fatty acids, methyl esters, and fatty alcohols (Biermann et al., 2011). To obtain specific chemicals of interest, subsequent chemical modification steps are often undertaken. These reactions primarily involve addressing the unsaturated bonds, such as epoxidation, hydroformylation, dimerization, thiol-ene coupling, oxidative cleavage (ozonolysis), olefin metathesis, pericyclic reactions, radical additions, as well as transition-metal catalyzed and Diels–Alder syntheses for aromatic compounds. The range of chemicals produced includes, but is not limited to, soaps, surfactants, emollients, fuel, pesticide formulations, and lubricants.

**FIGURE 3:**
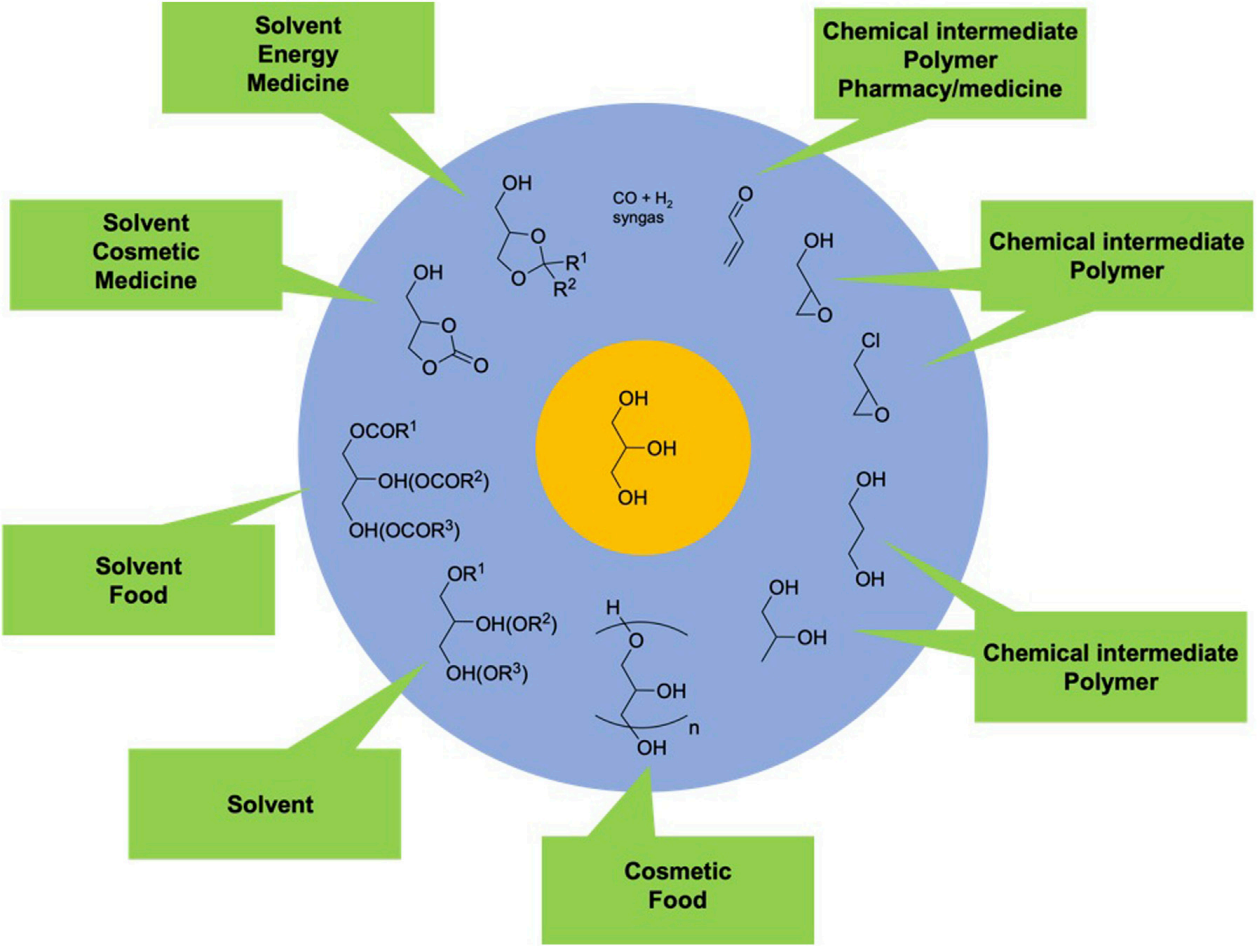
Roadmap to selected bio-based C_3_ chemicals starting from glycerol and their applications (Varma and Len, 2019).

Among the compounds obtained from vegetable oil, glycerol (propane-1,2,3-triol) is both a side-product of biodiesel industry and a platform molecule of interest (Len et al., 2018; Varma and Len, 2019; Zhao et al., 2023). With a steady compound annual growth rate (CAGR) of 1.7% projected between 2022 and 2031, the sales of glycerol are expected to rise from $4.3 billion to $5.1 billion (Glycerol market to Garner $5.1 billion, globally by 2031 at 1.7% CAGR says allied market research, 2022). The increasing demand for glycerol is driven by its wide-ranging applications in various products, including personal care items, pharmaceuticals, and the food and beverage industry. As a result, the demand for glycerol is anticipated to keep growing steadily. Moreover, glycerol is as a source of biobased C_3_ carbon atoms capable of being converted into more than 2000 products. These products include acrolein, acrylic acid, ethylene glycol, oxalic acid, syngas, 1,2- and 1,3-propanediol, mono-, di- and triglycerides, epichlorohydrin, polyglycerols, ketals, acetals, and many others. These derivatives find specific applications in industries like polymers, agrochemicals, and pharmaceuticals (Figure 3).

## 3 Mechanochemistry: principle, equipement and mechanism

Mechanochemistry has garnered significant interest as a powerful, sustainable, time-saving, environmentally friendly, and cost-effective synthesis method for preparing new functional materials. This approach relies on chemical and physicochemical transformations driven by mechanical force through grinding and milling. The IUPAC defines the mechanochemical synthesis as: “a chemical reaction that is induced by the direct absorption of mechanical energy” (Horie et al., 2004; Balaz et al., 2013; Rightmire and Hanusa, 2016). Mechanochemistry enables chemical reactions to take place through the application of mechanical energy, which can be provided either by manual grinding or automated milling, often without the use of solvents. When necessary, the use of small quantities of solvent is necessary to enhance chemical reactivity and this is where the term “Liquid-Assisted Grinding” (or solvent-drop grinding) is employed (Ying et al., 2021). When liquid-assisted grinding is used, the solubility of the reactants in the solvent is not considered because the solvent acts more like a catalyst. In order to determine whether we are in the domain of liquid-assisted grinding, the scale *η* has been defined as the volume of solvent (μL) divided by the total mass of the reactants (mg). The scale *?* varies from 0 < *η* < 1 and is independent of the nature of the solvent such as polarity, basicity, and hydrogen bonding capacity as well as the relative molar masses of the solvent and reactants.

**FIGURE 4:**
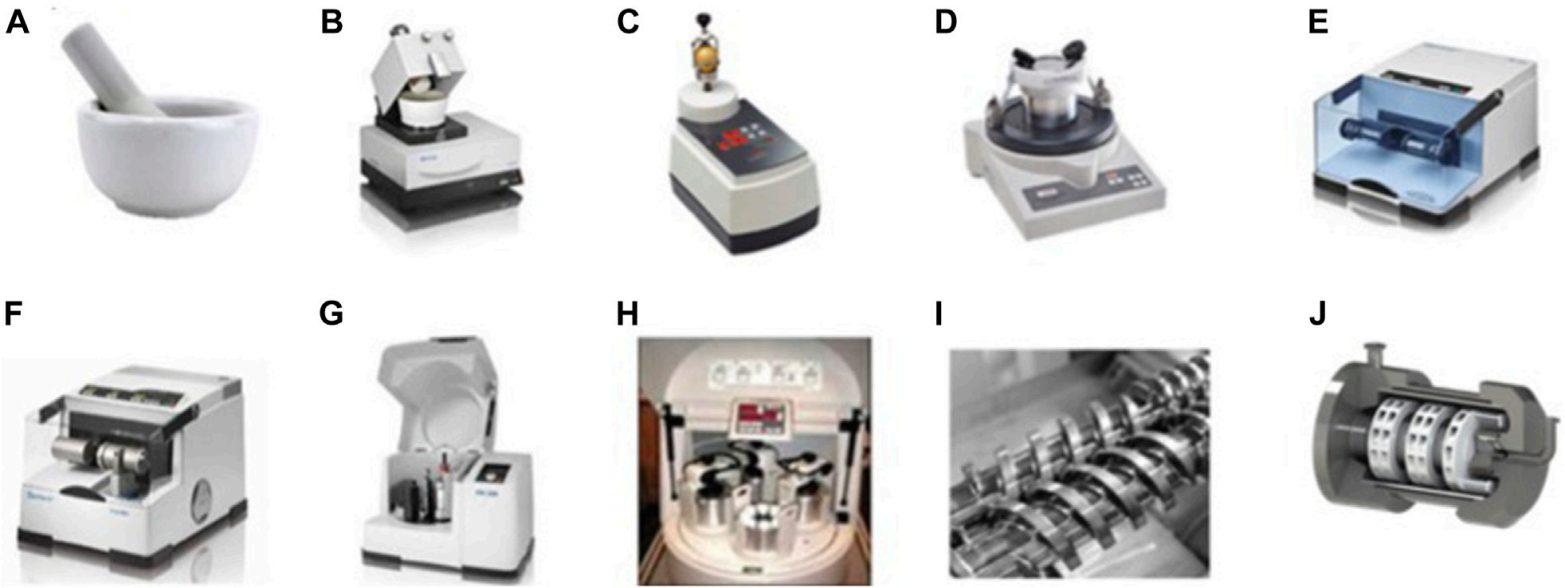
Most utilized types of grinding and milling equipment. **(A)** Mortar and pestle. **(B)** Automatic mortar. **(C)** Vertical vibrational mini-mill. **(D)** Vibratory micro-mill. **(E)** Vibrational ball mill. **(F)** Vibrational ball mill with temperature. **(G)** Planetary ball mill. **(H)** Multisample mill. **(I)** Twin screw for continuous mechanochemical synthesis. **(J)** Continuous flow heated mechanochemical reactor.

Manual grinding is commonly accomplished using a mortar and pestle. This equipment is simple to use and cost-effective, but it exposes the reagents to the environment, which can be hazardous. Alternatively, different automated milling devices are available for other grinding processes. For example, we can cite different batch reactors such as automatic mortars, vertical vibrational mini-mill, vibratory micro-mill, vibrational ball mill with or without temperature control, planetary ball mill, multisample mill (Figure 4). To date, only three types of continuous flow equipments exist: single and twin-screw for continuous mechanochemical synthesis and continuous flow heated mechanochemical reactor (Figure 4).

**FIGURE 5:**
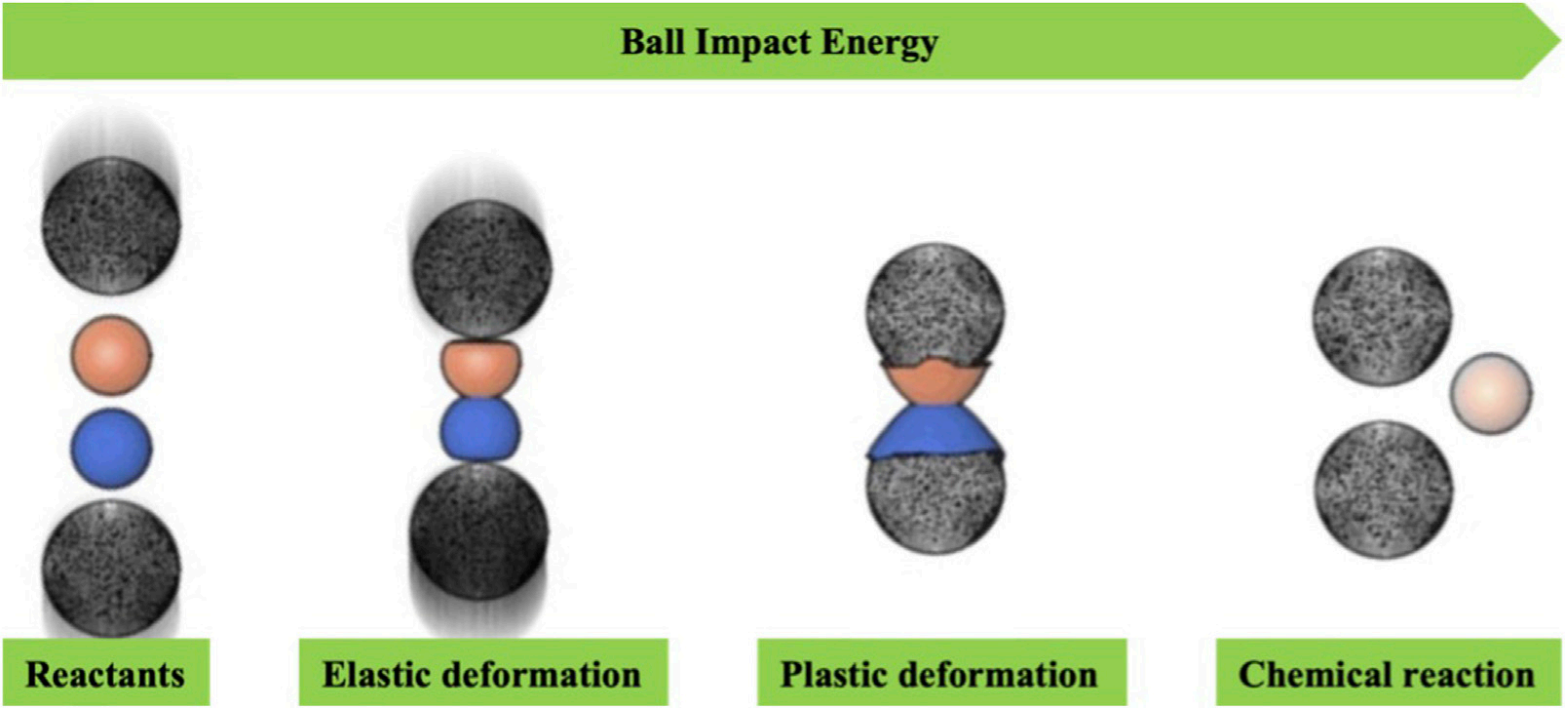
Basic concept for ball impact energy in mechanochemical synthesis (Wang et al., 2023).

The mechanism of mechanochemistry is still being studied; however, it is evident that mechanically activated reactions do not obey a single mechanism. Mechanochemistry through mechanical action (impact, shear, or friction) allows the breaking of intramolecular bonds, thereby generating subsequent chemical reactions (Michalchuk et al., 2021; O’Neill and Boulatov, 2021; Pagola, 2023). Impact is typically achieved through processes such as a jet mill, a falling hammer and disintegrator, while shear occurs in a mortar and pestle as well as an extruder. The simultaneous application of impact and shear forces is accomplished in devices such as a vibrational mill, an attritor, and a planetary mill (Figure 5). Regardless of the specific mode of action, the disruption of weaker bonds and the generation of surface plasmas resulting from mechanical impacts give rise to radical species. These species also play a role in activating chemical synthesis. The mechanical energy generated during mechanochemical activation leads to an increase in temperature, mass transfer, diffusion of surface molecules, reduction in powder size, and an increase in surface area. Various other mechanisms of action, each somewhat distinct, have been reported in the literature for mechanochemical synthesis. Among them, the friction-induced magma-plasma generation (hot spot theory) and the liquefaction of reagents through an amorphous phase or the formation of eutectic blends have been reported (Rothenberg et al., 2001; James et al., 2012).

Mechanochemistry finds applications across a diverse range of chemical disciplines, exemplified by cocrystals, metal–organic frameworks, molecular rearrangement, materials chemistry, polymer chemistry, porous materials, organic chemistry, biochemistry, pharmaceuticals, active pharmaceutical ingredients (APIs), and energy storage materials (Tan and Garcia, 2019; Cuccu et al., 2022). In the upcoming chapter, we will exclusively delve into the synthesis of small organic molecules utilizing oil-derived products such as triglycerides, fatty acids, fatty esters, glycerol and derivatives.

## 4 Mechanochemical transformations of oleochemicals into high-value small chemicals

### 4.1 From vegetable oils to biodiesel

**SCHEME 1:**
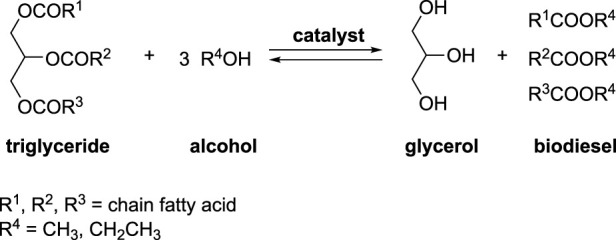
General chemical equation of the biodiesel formation.

Biodiesel presents a compelling alternative to petrodiesel, gaining increasing traction owing to growing environmental concerns and the drive to promote renewable energy. This sustainable fuel is derived from the transesterification of various vegetable oils, including edible, inedible, and residual ones, often employing methanol or ethanol. The resulting biodiesel is a blend of monoalkyl esters derived from long-chain fatty acids, ranging from C_8_ to C_24_ carbon atoms (Ma and Hanna, 1999). This mixture of fatty acid methyl esters boasts several environmental advantages, notably reducing emissions of CO, unburned hydrocarbons, particulates, and SO_2_. While both homogeneous and heterogeneous catalysts have been proposed, homogeneous catalysis is the preferred method for achieving a high yield of methyl/ethyl esters (Kiss et al., 2010; Borges and Diaz, 2012; Rizwanul Fattah et al., 2020). Homogeneous catalysis allows for the use of base catalysts (such as NaOH, KOH, NaOMe, KOMe) and acid catalysts (like H_2_SO_4_, HCl). Among these, base-catalyzed transesterification stands out as a more rapid and commercially favored option due to its operation under moderate conditions (Scheme 1). Nevertheless, a common challenge in biodiesel production, regardless of the catalyst used, is the poor miscibility of vegetable oils and methanol, leading to reduced reaction rates, especially in continuous flow processs. To address this limitation, alternative processes employing mechanochemistry in semi-continuous flow have been reported (Malpartida et al., 2020b; Malpartida et al., 2020). These innovative approaches offer potential solutions to enhance the overall efficiency of biodiesel production.

**SCHEME 2:**
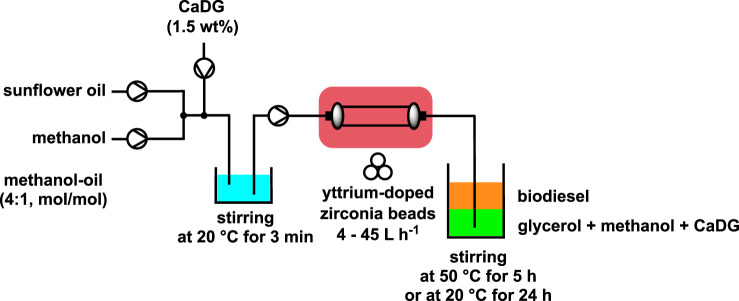
Semi-continuous biodiesel production catalyzed by CaDG using a high-throughput reactor filles with beads (Malpartida et al., 2020b; Malpartida et al., 2020).

CaO is a efficient base for the production of biodiesel (Alba-Rubio et al., 2010; Kouzu and Hidaka, 2012). According to some authors, CaO was the active phase only in the beginning of the reaction. Then, calcium diglyceroxide (CaDG) obtained from CaO and glycerol is the real catalyst of the trans-esterification (Kouzu and Hidaka, 2012; Kouzu et al., 2018). Using this concept, CaDG and triglyceride was studied for the formation of fatty acid methyl ester (FAME) *via* trans-esterification by employing a molar ratio of methanol to oil close to stoichiometry (4:1) and using 1.5% by weight of CaDG as the catalyst. With a continuous input flow between 4 and 45 L h^-1^ a semi-continuous mechanochemical reactor (volume of 0.5 L) containing yttrium-doped zirconia beads (diameter of 0.3–2.0 mm) occupying a 55%–70% volume was employed. Subsequent stirring for 4 h at 50°C or 24 h at room temperature completed the reaction and permitted to produce biodiesel with a yield of more than 90% (Scheme 2). This novel biodiesel production process is readily scalable and can be applied to the conversion of used cooking oils without any significant loss of yield (Malpartida et al., 2020b; Malpartida et al., 2020). A cost study was conducted, revealing the newfound approach to be more economical than the conventional batch stirred process. These results suggest that the methanol-oil molar ratio can be reduced from 12:1 to 4:1, and the catalyst weight can be decreased from 4% to 1.5% in a standard laboratory scale reactor, utilizing a more cost-effective process compared to the conventional batch stir method.

Mechanical energy, thermal energy, and pressure activation in continuous flow have proven highly advantageous in this biodiesel production process. The synergistic effect of achieving simultaneous activation has successfully addressed several drawbacks, leading to significant improvements, including: i) substantially shorter reaction times, ranging from hours to minutes; ii) lower operating temperatures attributed to the non-equilibrium conditions created by mechanical activation; iii) reduced mass transfer limitations due to an increased number of collisions; iv) minimized methanol usage; v) enabled real-scale production, transitioning from milliliters to liters; vi) increased yields and/or selectivity, owing to the simultaneous regeneration of the catalyst surface during the reaction.

The same research group investigated biodiesel production from both sunflower oil and used cooking oil, employing a slightly higher molar ratio of methanol to oil (5:1 compared to 4:1) and a lower flow rate (4 L h^-1^) (Malpartida et al., 2020b; Malpartida et al., 2020). The findings indicated that similar catalytic activity was observed up to 120 min, yielding 80%. However, beyond 120 min, the ester yields from the oils decreased by 17%. The authors propose that this reduction may be attributed to the neutralization of the basic sites of the calcium diglycerate (CaDG) catalyst by the free fatty acids present in the waste oils.

### 4.2 From fatty acids methyl ester to lipidyl-cyclodextrins

**SCHEME 3:**
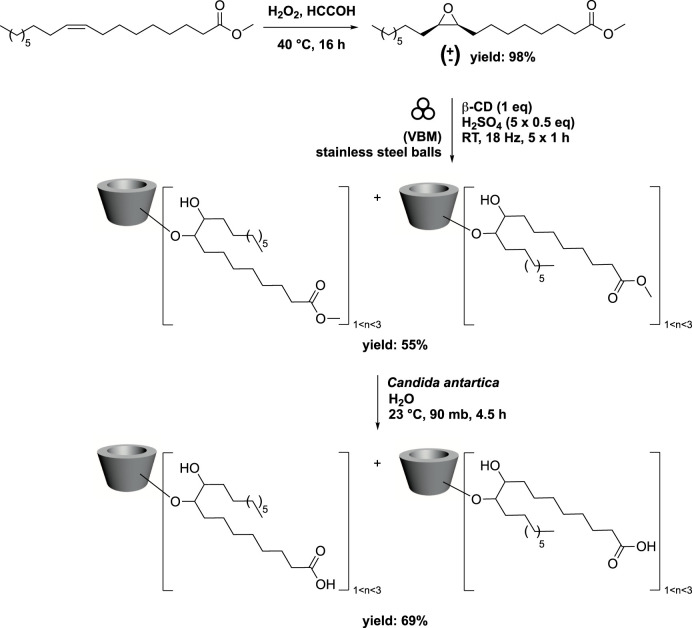
Synthesis of bicaternary lipidyl-cyclodextrins from oleic acid in 3 steps *via* ring-opening of methyl oleate epoxide by vibrating ball-milling in a mixer mill (Oliva et al., 2020).

Cyclodextrins (CDs) are macrocyclic malto-oligosaccharides produced from starch through enzymatic conversion. They consist of α-(1→4)-linked D-glucopyranose units in a ^4^C_1_ chair conformation. Due to their unique 3D structure, unmodified CDs are hydrophilic with a conical cavity that is essentially hydrophobic. This characteristic enables CDs to form inclusion complexes with various lipophilic guest molecules, utilizing their hydrophobic cup-like structure (Szejtli, 1998). By introducing lipophilic groups, modified CDs gain self-assembly properties and the ability to form complexes with invited molecules, enhancing their vectorization capabilities. In this context, bicaternary lipidyl-cyclodextrins have been synthesized by combining methyl oleate epoxide with various CDs (Oliva et al., 2020). The process began with conventional epoxidation of methyl oleate using H_2_O_2_ in the presence of formic acid, yielding a racemic mixture of the corresponding methyl oleate epoxide. To prevent the formation of a sticky paste, which could hinder reactivity, the epoxide’s ring opening was conducted in five steps. Successive additions of methyl oleate epoxide and H_2_SO_4_ were made to the β-CDs, and the reaction was carried out by vibrating ball-milling (VBM) in a mixer mill (volume of 65 mL) containing 12 stainless steel balls (3 balls with diameter of 12.7 mm, 5 balls with diameter of 6.4 mm, and 4 balls with diameter of 4.0 mm). For the balls, the use of different diameters and the ratio between them were not studied. The ball milling process was conducted for 1 h at a frequency of 18 Hz. The target compound was obtained in 55% yield, with 1, 2, or 3 fatty acid methyl ester grafts. Next, enzymatic hydrolysis of the terminal ester was performed in the presence of *Candida antarctica* in water at 90 mbar and 23 °C, resulting in the corresponding carboxylic acid in 69% yield (Scheme 3).

In order to have a structure activity relationship, the authors tried different CDs with or without hydroxypropyl groups (α-CD, β-CD, γ-CD, HP-α-CD, HP-β-CD, HP-γ-CD) permitting to obtain α-CD (C_9_)_2_OOMe, γ-CD (C_9_)_2_OOMe, HP-α-CD (C_9_)_2_OOMe, HP-β-CD (C_9_)_2_OOMe and HP-γ-CD (C_9_)_2_OOMe in 28%–44% yields.

### 4.3 From fatty acids to dimeric fatty acids

The oleochemical dicarboxylic acids make up approximately 0.5% of the total dicarboxylic acid market for monomers, with phthalic and terephthalic acids representing 87%. Due to their unique chemical nature, these oleochemical-derived dicarboxylic acids tend to modify or alter condensation polymers, thus occupying a special niche market. Some desirable properties associated with oleochemical-derived dicarboxylic acids include elasticity, flexibility, high impact strength, hydrolytic stability, hydrophobicity, lower glass transition temperatures, and increased flexibility (Paraskar et al., 2023).

**SCHEME 4:**
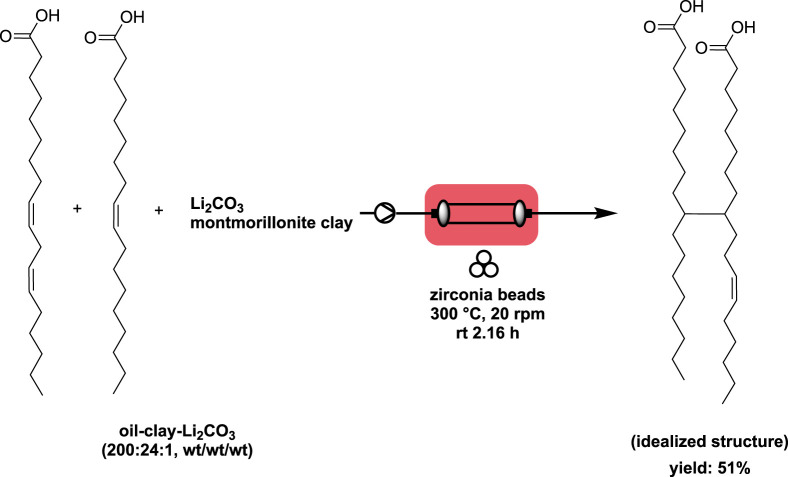
Continuous dimerization of soybean fatty acid using a horizontal ball-mill reactor (Lu et al., 2019).

Researchers reported the continuous production of dimeric fatty acid from soybean fatty acid (oleic acid-linoleic acid weight ratio of 33:65) in the presence of acidic montmorillonite clay and lithium carbonate (Lu et al., 2019). To facilitate the reaction, an innovative ball-mill reactor was designed and utilized for this study. The stainless reactor had 3 baffles to enhance radial turbulence and 10 clapboards to divide the reactor into 11 small reactors in series, making it akin to a plug-flow reactor. A solution of oil-clay-lithium carbonate with a weight ratio of 200:24:1 was injected using a peristaltic pump into the horizontal continuous ball mill reactor, which was filled with zirconia beads (bead-to-mass ratio = 12) and operated at 300°C. The residence time was 2.16 h, and the rotation speed was set at 20 rpm. The resulting dimeric fatty acid was obtained with a yield of 51% (Scheme 4). Interestingly, the authors found that using similar conditions in a stirred flask (500 mL) at 400 rpm allowed for the production of the diacid with a yield of 63%. However, the lower yield obtained in the continuous flow process was attributed to the dissolution of some metal ions, such as Fe^3+^ and Ni^2+^, from the continuous ball mill reactor.

### 4.4 From natural fatty acids to labeled fatty acids

Fatty acids play a crucial role in biological systems and have wide-ranging applications in materials science, including drug formulation and surface-functionalization of nanoparticles (Magkos and Mittendorfer, 2009; Remize et al., 2020). To gain a deeper understanding of their biological mechanisms, researchers have reported the mechanochemical synthesis of isotopically labeled fatty acids, specifically with ^17^O and ^18^O labeling.

**SCHEME 5:**
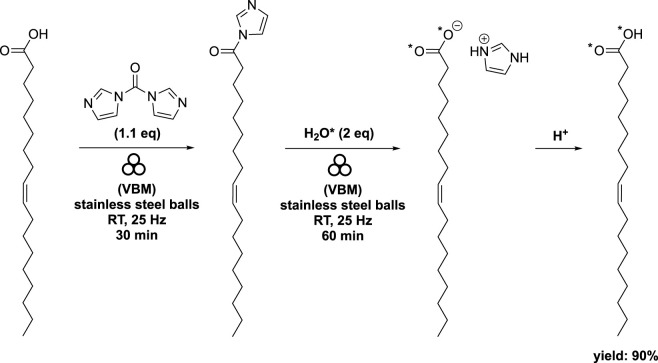
Synthesis of labeled oleic acid by vibrating ball-milling in a mixer mill (Spackova et al., 2020).

The target compounds were successfully produced through two mechanochemical steps: first, by activating the carboxylic function using 1,1′-carbonyldiimidazole (CDI), and then hydrolyzing the acyl imidazole intermediate with enriched H_2_O* (97% ^18^O-enriched H_2_O* and 90% ^17^O-enriched H_2_O*) (Spackova et al., 2020). The synthesis process involved milling a mixture of oleic acid and CDI (1.1 eq) at 25 Hz for 30 min using vibrating ball-milling (volume of 10 mL) in a mixer mill filled with two stainless steel beads (diameter of 10 mm). Subsequently, complete hydrolysis with H_2_O* (2 eq) was performed for 1 h using the same equipment. After standard work-up procedures, the target compound was obtained with an impressive 90% yield, with an average enrichment of 44% for the ^18^O labeled molecules and 36% for the ^17^O labeled molecules per carboxylic oxygen (Scheme 5).

**SCHEME 6:**
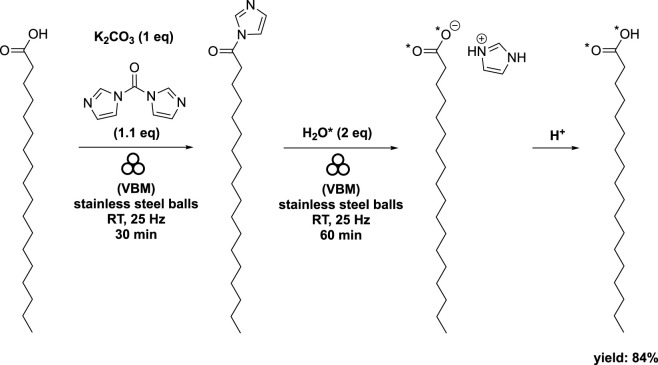
Synthesis of labeled stearic acid by vibrating ball-milling in a mixer mill (Spackova et al., 2020).

When stearic acid was used instead of oleic acid, the addition of K_2_CO_3_ (at 1 eq) as a base was necessary to expedite the hydrolysis step (Scheme 6). The authors explained that the base promoted the formation of HO*- and subsequently facilitated the deamidation reaction. Interestingly, the appearance of the mixture varied when starting from oleic acid compared to stearic acid. The acyl imidazole intermediate derived from oleic acid had a pasty consistency, while that derived from stearic acid exhibited a more powdery texture (Spackova et al., 2020).

**FIGURE 6:**
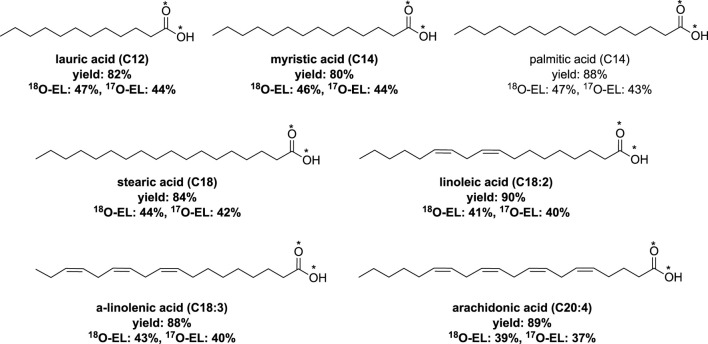
Synthesis of labeled fatty acids by vibrating ball-milling in a mixer mill (Spackova et al., 2021).

An extension of this process was reported by the same research group, which involved fatty acids with a carbon chain containing carbon atoms between C12 to C18 (Spackova et al., 2021). Additionally, the extension encompassed polyunsaturated fatty acids (PUFAs) containing up to four double bonds. The hydrolysis time varied depending on the acyl imidazole derivatives used, ranging from 1 to 3 h (Figure 6).

**FIGURE 7:**
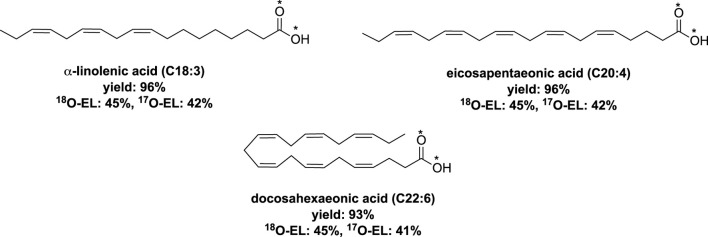
Labeled polyunsaturated fatty acids obtained by vibrating ball-milling in a mixer mill (Spackova et al., 2021).

The researchers also developed a one-pot mechanochemical saponification method for other polyunsaturated fatty acids (PUFAs), namely, ethyl α-linolenate, ethyl eicosapentaenoate, and ethyl docosahexaenoate (Spackova et al., 2021). In this process, a mixture of the ethyl ester of the respective PUFA, sodium ethoxide (at 1.5 equiv.), and H_2_O* (at 3 eq) was milled at 25 Hz for 30 min using vibrating ball-milling in a mixer mill. Employing conventional work-up techniques, the corresponding labeled PUFAs were obtained with yields exceeding 93% and showed high enrichment levels (Figure 7).

### 4.5 From glycerol to calcium diglyceroxide (CaDG)

As mentioned earlier for biodiesel production, CaDG serves as an active basic catalyst and is commonly synthesized through conventional methods (Kouzu and Hidaka, 2012; Kouzu et al., 2018; Malpartida et al., 2020b; Malpartida et al., 2020). Additionally, CaDG finds applications as a plasticizer and thermal stabilizer for plastics. Seeking an alternative process for CaDG production, a mechanochemical approach involving a mixture of glycerol and CaO in a 1:5 M ratio was reported (Lukic et al., 2016). They did a planetary ball milling (PBM) operating in an air atmosphere at 270 rpm for 5 h. Afterward, methanol was added, and the resulting mixture was further milled for 15 min, followed by conventional work-up. This procedure allowed the synthesis of CaDG in high yield. The catalyst thus obtained was then utilized for the methanolysis of sunflower oil, resulting in the corresponding FAME (fatty acid methyl esters) with high efficiency.

Subsequently, the synthesis of CaDG using a continuous mechanochemical reactor (MCR), utilizing the same equipment employed for biodiesel production (Malpartida et al., 2020b; Malpartida et al., 2020). Glycerol and CaO were mixed in two different molar ratios, 3 and 5, in a mixer tank. The resulting mixture was pumped into the continuous mechanochemical reactor (volume of 0.5 L) at a flow rate of 4 and 150 L h^-1^, respectively. The reactor was filled with yttrium-doped zirconia beads (diameter: 0.3–2.0 mm), occupying 55%–70% of the reactor volume. In this study, the quantification of crystalline CaDG formation was accomplished through the utilization of the Rietveld method. An X-ray diffraction (XRD) analysis was performed on all the samples, and the nominal composition derived from the structure identification step was refined using a Rietveld analysis. The most accurate refinement was achieved by considering factors such as sample displacement, background correction, peak profile parameters, and the scaling of each distinct phase. Using yttrium-doped zirconia beads (diameter of 0.5 mm), CaDG was obtained with quantitative yield after a 30-min residence time (Table 1, lines 6–9). However, with a glycerol-CaO molar ratio of 3, the temperature needed to be increased to 50°C for optimal yield (Table 1, lines 5 and 6). At room temperature (25°C), a higher glycerol-to-CaO molar ratio 5) was necessary to achieve yields exceeding 99% (Table 1, lines 5 and 7). At 50 °C, the same conditions gave only CaDG in 55% yield. The researchers also tested the influence of water presence due to glycerol’s hydrophilic nature. In the presence of 10 wt% water and a glycerol-CaO molar ratio of 5, CaDG was obtained in quantitative yield with a residence time of 30 min (Table 1, entry 9).

**TABLE 1:** Influence of different experimental variables on the formation of CaDG using a continuous mechanochemical reactor (Malpartida et al., 2020b; Malpartida et al., 2020). *^a^*

Entry	Glycerol quality (wt% of H_2_O)	rt (min)	Glycerol-CaO molar ratio	T (°C)	Yield of CaDG (XRD) (%)
1	0	5	3	25	15
2	0	5	3	50	18
3	0	5	5	25	18
4	0	5	5	50	84
5	0	30	3	25	91
6	0	30	3	50	>99
7	0	30	5	25	>99
8	0	30	5	50	55
9	10	30	5	50	>99

^a^ Reaction conditions: a mixture of glycerol-CaO, molar ratio (3 or 5) was pumped into the continuous mechanochemical reactor filled with yttrium-doped zirconia beads (diameter of 0.5 mm), occupying 55%–70% of the reactor volume for a residence time (5 and 30 min).

**SCHEME 7:**
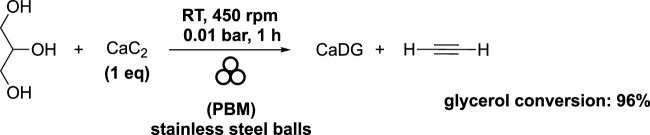
Synthesis of acetylene and CaDG starting from calcium carbide and glycerol in a planetary ball mill (Li et al., 2018).

CaDG was obtained as a side-product during the mechanochemical production of acetylene by reacting calcium carbide (CaC_2_) with glycerol (Li et al., 2018). The molar ratio of glycerol-CaC_2_ used was 1, and the mixture was subjected to 1 hour of milling at 450 rpm at room temperature in a planetary ball mill (volume of 250 mL) filled with stainless steel balls. The stainless steel balls comprised 2 balls with a diameter of 15 mm, 4 balls with a diameter of 12 mm, 25 balls with a diameter of 10 mm, 43 balls with diameters of 08 mm, and 232 balls with a diameter of 3 mm. As previously reported, the choice to use of different diameters and different ratio between them were not described by the authors. To prevent air impurities, a vacuum of 0.01 bar was applied, which also minimized the risk of explosions due to the presence of oxygen. As a result, the desired acetylene was obtained in good yield, with 96% glycerol conversion (Scheme 7).

The authors highlighted that the mechanochemical reaction rate of CaC_2_ with glycerol significantly increased with the rising rotating speed, and the efficiency of this reaction exceeded that of the corresponding thermal chemical reaction. In summary, the current process proves effective for the efficient production of high-purity acetylene and CaDG. The mechanochemical reaction of CaC_2_ with glycerol demonstrates greater efficiency than the corresponding thermochemical reaction at high temperatures, with CaC_2_ showing higher reactivity compared to CaO with glycerol. Importantly, when compared to conventional acetylene production methods, the authors did not detect any PH_3_, AsH_3_, and H_2_S formed as side reactions involving Ca_3_P_2_, Ca_3_As_2_, and CaS, respectively. This absence is attributed to the higher reactivity of glycerol relative to water, which enhances the reaction’s selectivity.

### 4.6 From glycidol to glycerol esters

Esterification of glycerol enables the production of various valuable chemicals, such as monoacylglycerol (MAG) and diacylglycerol (DAG). Among these chemicals, diacylglycerols (DAGs) play significant signaling roles in cells (DAG signaling), along with many other amphipathic or hydrophobic small molecules exhibiting lipid structures (Eichmann and Lass, 2015). DAGs are glycerolipids composed of two fatty acids esterified to glycerol. DAGs are produced through the enzymatic breakdown of glycerophospholipids and the lipolysis of TAGs (triacylglycerols). Extracting pure DAGs and TAGs from natural sources proves challenging due to the structural diversity of fatty acids in acylglycerols and their subtle differences in chain length, degree of unsaturation, double bond position, and stereochemistry. The chemical synthesis of protected DAGs from glycerol or glycidol requires several preparative steps involving organic solvents like CH_2_Cl_2_, THF, or Et_2_O (Martin et al., 1994).

**SCHEME 8:**
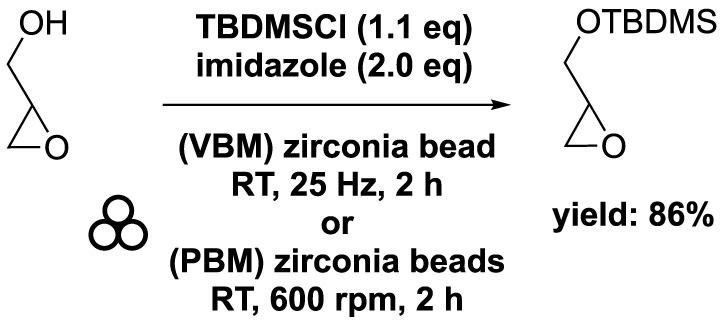
Silylation of glycidol with TBDMSCl by vibrating ball-milling in a mixer mill or in a planetary ball mill (Ardila-Fierro et al., 2019).

To achieve greener conditions and produce MAG and DAG derivatives, researchers have investigated solventless mechanochemical synthesis. In this process, a mixture of glycidol, tert-butylchlorodimethylsilane (1.1 eq), and imidazole (2.2 eq) was combined in a mixer mill (volume of 10 mL) filled with one zirconia ball (diameter of 10 mm) and subjected to 25 Hz for 2 h. The resulting tert-butyl-dimethylsilyl glycidyl ether was obtained with an 86% yield (Ardila-Fierro et al., 2019). Similarly, a comparable experiment using a planetary ball mill at 600 rpm yielded the target chemical in an 88% yield, demonstrating no significant difference between the two equipment setups (Scheme 8). Comparison with conventional process showed that the ball milling approach facilitated a solventless reaction and reduced the reaction time from overnight to just 2 h (Zaed and Sutherland, 2011; Winkler et al., 2013).

**SCHEME 9:**
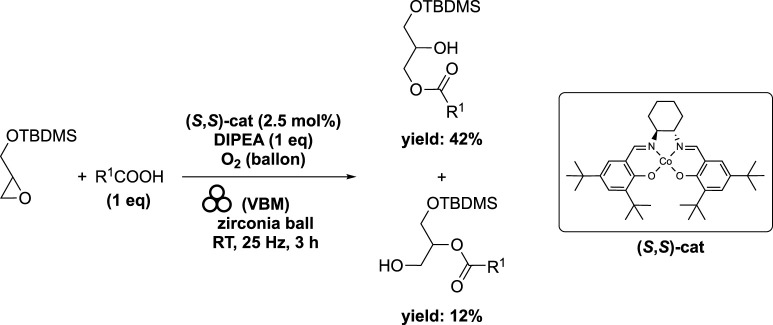
Cobalt-catalyzed ring-opening of epoxide by vibrating ball-milling in a mixer mill (Ardila-Fierro et al., 2019).

In order to have a good selectivity for the ring opening of the epoxide with fatty acid as nucleophile, the use of Jacobsen cobalt (II)-salen complex (*S*,*S*)-cat has been selected. A blend of stearic acid (1 eq) and chiral (*S*,*S*)-cat (2.5 mol%) was subjected to milling in a mixer mill (volume of 20 mL) containing a single zirconia ball (diameter of 10 mm) for 15 min at 25 Hz under an oxygen atmosphere. Subsequently, DIPEA (1 eq) was introduced, and the resulting mixture was milled for an additional 10 min. Lastly, glycidyl ether (1 eq) was added to the container, and the resulting mixture was milled at 25 Hz for 155 min. This process yielded two regioisomers: 1-stearoyl-3-(*tert*-butyldimethylsilyl)-sn-glycerol in 42% yield and 2-stearoyl-3-(*tert*-butyldimethylsilyl)-sn-glycerol in 12% yield (Ardila-Fierro et al., 2019) (Scheme 9). Compared to the solventless batch reaction, the ball milling process reduced the reaction time from 16 h to 3 h (Fodran and Minnaard, 2013). It is worth noting that the initial 16-h duration was not an optimized experimental condition.

**SCHEME 10:**
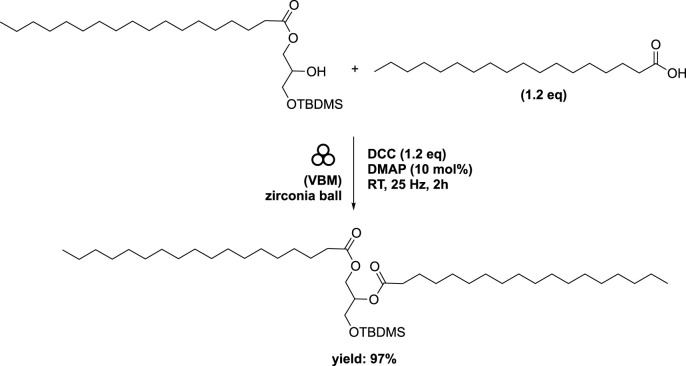
Mechanical synthesis of DAG obtained by vibrating ball-milling in a mixer mill (Ardila-Fierro et al., 2019).

**FIGURE 8:**
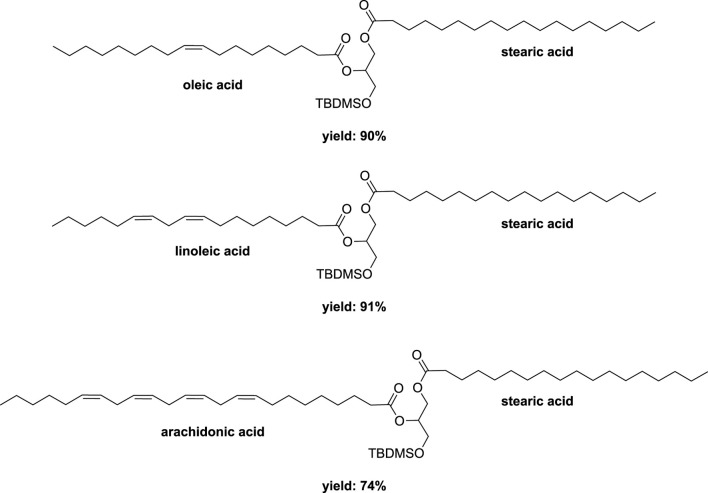
Different DAGs obtained by vibrating ball-milling in a mixer mill (Ardila-Fierro et al., 2019).

Subsequently, starting with the major regioisomer 1-stearoyl-3-(*tert*-butyldimethylsilyl)-sn-glycerol, a secondary esterification was investigated using stearic acid (1.2 eq) along with DCC (1.2 eq) and DMAP (10 mol%) in a mixer mill (volume of 10 mL) containing a single zirconia ball (diameter of 10 mm) at 25 Hz for 2 h. This procedure afforded diester in a remarkable 97% yield (Scheme 10). Application of this method to oleic acid, linoleic acid, and arachidonic acid yielded the corresponding diesters in 90%, 91%, and 74% yields, respectively (Figure 8). While similar conditions were employed in conventional reactions conducted in heptane, the time required was substantially longer (16 h compared to 2 h), despite the absence of any optimizations (Fodran and Minnaard, 2013). The subsequent deprotection of the primary hydroxyl group to remove the silyl group was accomplished under acidic conditions, yielding the 1,2-distearoyl derivative in an 81% yield. In order to achieve higher yields and prevent acyl migration from the secondary to primary positions, the ball milling technique was not employed.

**SCHEME 11:**
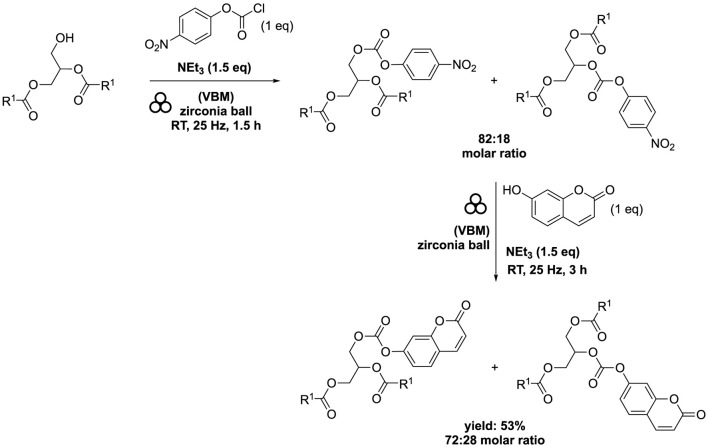
Conjugation of DAG with 7-hydroxycoumarin by vibrating ball-milling in a mixer mill (Ardila-Fierro et al., 2019).

The diester 1,2-distearoyl-3-(*tert*-butyldimethylsilyl)-sn-glycerol was combined with 4-nitrophenyl chloroformate (1 eq) in the presence of NEt_3_ (1.5 eq). Subsequently, the resulting mixture underwent milling using the same equipment, operating at a frequency of 25 Hz for 90 min. Without the need for additional purification steps, the reaction mixture was then introduced to 7-hydroxycoumarin (1 eq) and NEt_3_ (1.5 eq). The combined mixture was stirred at 25 Hz for 3 h. This process yielded a complex mixture of two compounds: 1,2-distearoyl-3-((((2-oxo-2H-chromen-7-yl)oxy)carbonyl)oxy)-sn-glycerol and 1,3-distearoyl-2-((((2-oxo-2H-chromen-7-yl)oxy)carbonyl)oxy)-sn-glycerol, in a molar ratio of 2.5:1. The overall yield achieved from this procedure was 53% (Scheme 11). A acyl migration occurred from the secondary hydroxyl group to the primary hydroxyl group as expected but this migration was limited.

### 4.7 From glycerol to benzoxazine

Benzoxazines are heterocyclic compounds with potent biological activities (Garg et al., 2013) as monomer and with unique properties after polymerization. Polybenzoxazine is a relatively new class of polymeric phenolic resins with various excellent characteristics, including outstanding stiffness, low dielectric constant, high heat resistance, low flammability, and minimal moisture absorption when exposed to moisture, chemicals, and other corrosive liquid, therefore, they are ideal polymers to use in special and extreme cases (Machado et al., 2021; Ding et al., 2022).

In order to produce a biobased benzoxazine, a mixture of glycerol (1–3 eq), diethyl carbonate (1.4 eq), and 2-aminophenol (1 eq) was combined with K_2_CO_3_ (0.1 eq). This mixture was then processed within a planetary ball mill (volume of 125 mL) filled with stainless steel balls (60 g with a diameter of 2 mm) for durations of 60 and 120 min operating at 350 rpm. The outcomes of these experiments underscore that mechanochemistry is not a miraculous technology. Attempts to use liquid-assisted grinding by introducing a small quantity of acetone were unsuccessful (Torres-Pastor et al., 2022). Additional tests were conducted to initiate the reaction utilizing microwaves as an alternative technology. These experiments yielded improved conversions (<25%) and selectivities (<35%), albeit still at a relatively low level.

**SCHEME 12:**
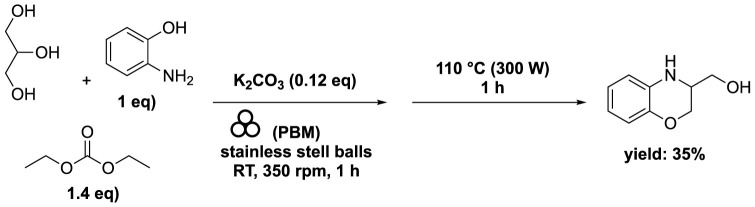
Synthesis of benzoxazine from glycerol using successive mechanochemistry in ball mill and microwave irradiation (Torres-Pastor et al., 2022).

Recent studies have shown promising advancements in certain reactions through a combination of successive milling and microwave techniques (Martinez et al., 2023). In one experiment, glycerol (1 eq), diethyl carbonate (1.4 eq), 2-aminophenol (1 eq), and K_2_CO_3_ (0.12 eq) were subjected to milling in a planetary ball mill reactor (volume 125 mL), containing stainless steel balls (60 g with a diameter of 2 mm), for 60 min at 350 rpm. Subsequently, a microwave-assisted organic reaction was conducted at 110°C (300 W) for 60 min. This process led to a 38% conversion of 2-aminophenol, with a remarkable 93% selectivity towards the benzoxazine derivative (Torres-Pastor et al., 2022) (Scheme 12). Increase the rpm from 350 to 1,000 to enhance the phenol conversion, while significantly reducing the selectivity from 93% to 7%. Notably, attempts at conventional heating in place of microwave activation resulted in a conversion of 2-aminophenol of less than 5%.

### 4.8 From glycerol to solketal

**SCHEME 13:**
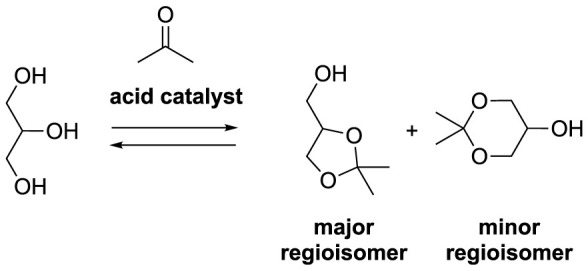
General ketalization of glycerol with acetone in acid conditions.

Solketal (1,3-di-*O*-isopropylidene-glycerol) holds significant promise across various domains, serving as a versatile molecule in pharmaceutical chemistry as a synthetic intermediate, an isopropylidene protective group in C3-based organic chemistry, a solvent and plasticizer in polymer chemistry, and even as a fuel additive (Garcia et al., 2008; Mota et al., 2010; Silva et al., 2010; Nanda et al., 2016; Zahid et al., 2021; Yu et al., 2022). The synthesis of solketal involves an acetalization reaction between glycerol and acetone under acidic conditions (Zahid et al., 2021). This reaction exists in equilibrium, potentially yielding 1,3-di-*O*-isopropylidene-glycerol as a byproduct (Scheme 13). Typically, homogeneous acid catalysts such as H_2_SO_4_ or HCl are employed, although both homogeneous Lewis acids and heterogeneous acid catalysts can also find application (Kiakalaiehet et al., 2018; Zahid et al., 2020).

Given the industrial outlook, extensive efforts have been directed towards developing continuous flow processes for solketal synthesis (Khodadadi et al., 2021). However, certain limitations persist within these continuous flow methods. Challenges include the immiscibility of glycerol with acetone due to differing physicochemical parameters, necessitating the use of organic co-solvents. Additionally, homogeneous catalysis demands substantial quantities of catalysts, while heterogeneous catalysis suffers from low flow rates. The presence of water, a byproduct of the reaction, also leads to catalyst deactivation.

In addition to the transfer of mechanical energy and milling, bead mechanochemistry emerges as a highly efficient method for homogenization. Indeed, patented studies have demonstrated that this innovative technique effectively addresses the miscibility challenges encountered in solketal synthesis under homogeneous acid catalysis conditions (Len et al., 2021). This method has been employed for the continuous flow mechanochemical synthesis of solketal, using equipment and processes similar to those described above for CaDG and continuous flow biodiesel mechanochemistry (Malpartida et al., 2020b; Malpartida et al., 2020). Various conditions and homogeneous acid catalysts were examined, with the most favorable outcomes observed for FeCl_3_•6H_2_O as Lewis acid in both loop and continuous flow processes at 56°C. This led to a remarkable 99% solketal yield within residence times of less than 15 min. These investigations in presence of a solvent stand as pioneering examples showcasing the efficacy of continuous flow bead mechanochemistry in facilitating organic synthesis.

### 4.9 Advantages and limits

**FIGURE 9:**
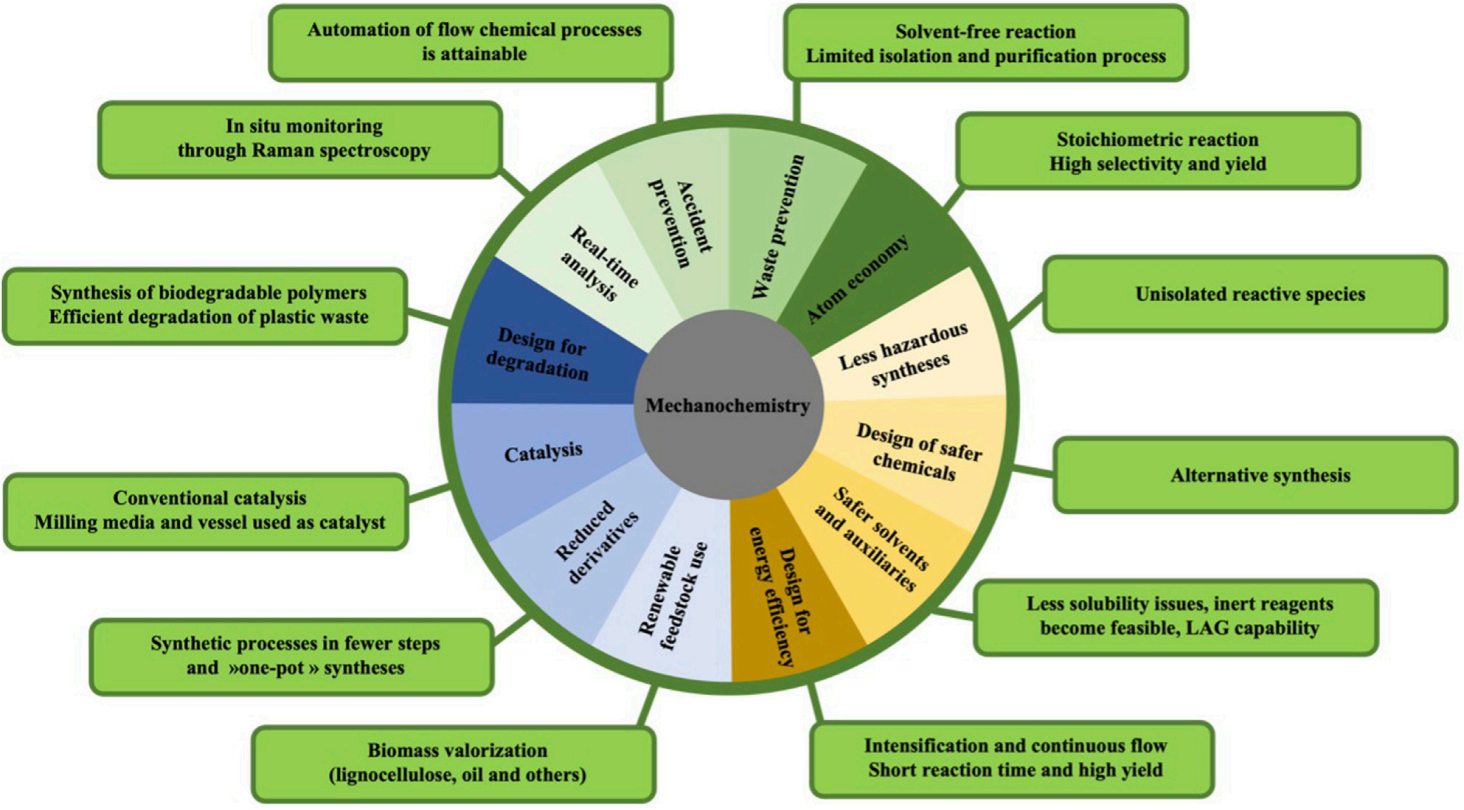
Twelve principles of green chemistry and mechanochemistry inputs.

Mechanochemistry has become an attractive method for producing various materials, polymers and small chemicals. Most of these advantages can be associated with the twelve principles of green chemistry (Figure 9).

The main advantages of mechanochemistry encompass the promotion of solvent-free reactions, leading to a significant reduction in waste and pollution generation while simultaneously lowering economic costs. Additionally, mechanochemical processes often yield larger quantities of products when compared to analogous solution reactions. They also generally entail shorter reaction times, meticulous control over stoichiometry, and enhanced product selectivity. Another notable advantage of mechanochemistry, as opposed to traditional solution-based methods, is its capability to enable the utilization of reagents that are conventionally deemed inert or highly insoluble. However, it is crucial to acknowledge that mechanochemistry does not provide a universal solution to all synthetic challenges. Although mechanochemistry is an ancient technique, the understanding of the entire range of phenomena is still in its early stages, particularly concerning fundamental physicochemical knowledge. Indeed, to this day, the thermodynamic variables used to study and control chemical reactions, such as temperature and pressure, are not yet measured or controlled systematically. Other parameters and mechanisms are still unknown: the amounts of energy transferred or made available through the combination of temperature and applied mechanical treatment, mass transfers in heterogeneous phase reactions, increased selectivity, and more. In summary, mechanochemistry offers environmentally friendly and efficient reactions but is typically restricted to solid-state processes. In contrast, oleochemistry provides sustainable and biodegradable chemicals, both in liquid and solid forms. Both fields come with their distinct advantages and challenges, making them valuable in diverse applications within the chemical industry.

## 5 Conclusion

This comprehensive review explores the significant contributions of oleochemistry, emphasizing its vital role in providing non-fossil resources and invaluable molecules. It also delves into the growing interest in combining mechanochemistry with molecules derived from untreated or modified oils, guided by insights from relevant scientific studies. Oleochemistry, which includes vegetable oils and their derivatives, offers a wealth of platform molecules for producing high-value chemicals, presenting a promising alternative to petroleum resources. The diverse chemical components within oleochemicals, such as esters, C-C double bonds, and hydroxyl groups, make them versatile building blocks. Mechanochemical synthesis emerges as an environmentally conscious, potent, sustainable, time-efficient, cost-effective, and ecologically friendly method for creating novel functional materials. By manipulating various synthetic parameters, such as milling methods, materials, time, temperature, and process control agents, biobased molecules’ synthesis can be significantly improved compared to traditional activation methods. Mechanochemistry has widespread applications across different research areas and industries, making it crucial for future investigations.

The review highlights key achievements in mechanochemistry and oleochemistry. Notably, a semi-continuous mechanochemical reactor equipped with beads achieved a biodiesel yield exceeding 90% from triglycerides, showcasing scalability and environmental benefits. Mechanochemical reactions starting from fatty acids and esters, including the use of methyl oleate epoxide and cyclodextrins, demonstrated the technique’s merits. Continuous production of dimeric fatty acids in a ball-mill reactor and the synthesis of labeled fatty acids further illustrate its potential. Glycerol, another essential component in oleochemistry, was successfully utilized in various mechanochemical reactions. A continuous mechanochemical reactor with beads produced CaDG compounds with exceptional selectivity and high yields. The synergy of glycerol and CaC2 in a planetary ball mill resulted in acetylene production of superior quality. The review also covers the synthesis of various glycerol-based compounds, demonstrating the versatility of glycerol in mechanochemistry. The text acknowledges that, while comparisons between conventional and mechanochemical methods are sometimes made, these often involve variations in reagents and the presence of solvents, making it challenging to assess the distinct contributions of different milling technologies.

In summary, this review underscores the pivotal role of oleochemistry in providing sustainable and versatile molecules while showcasing the potential of mechanochemistry for the environmentally conscious and efficient synthesis of biobased compounds. The achievements in biodiesel production, fatty acid transformations, glycerol-based reactions, and other applications exemplify the exciting possibilities in this interdisciplinary field.
